# Endoscopy and histology in inflammatory bowel diseases patients: Complementary or alternatives?

**DOI:** 10.1002/ueg2.12202

**Published:** 2022-01-22

**Authors:** Ferdinando D'Amico, Alessandra Zilli, Gionata Fiorino

**Affiliations:** ^1^ Gastroenterology and Endoscopy IRCCS Ospedale San Raffaele and Vita‐Salute San Raffaele University Milan Italy; ^2^ Department of Biomedical Sciences Humanitas University Milan Italy

To date, endoscopic remission is the main therapeutic target in patients with ulcerative colitis (UC), influencing treatment decisions.^1^ However, growing evidence shows that subjects with endoscopic remission but persistent histological activity have an increased risk of clinical recurrence.^2^ Furthermore, histological disease activity has been associated with an increased risk of hospitalization and surgery, and it has been identified as an independent risk factor for the use of systemic corticosteroids in patients with endoscopic remission.^3^, ^4^ The attempt to achieve the deepest disease remission possible has led to the new concept of disease clearance, defined as simultaneous clinical, endoscopic, and histological remission. Patients who achieve disease clearance are more likely to have better prognosis than those who do not.^5^ These data suggest that endoscopic remission is probably not the ultimate target, if not combined with histological remission too. In the recent years, the introduction of high‐definition white light colonscopy and virtual chromoendoscopy (VCE) has brought a significant advance in the management of UC, as they allow to obtain a better visualization of mucosal surface and vascular pattern.^6^ The Paddington International Virtual Chromoendoscopy Score (PICaSSO) is a new endoscopic VCE score calculated through the i‐SCAN technique (Pentax), which allows to predict the histological activity of disease, without the use of biopsies.^7^ A recent study by Iacucci et al. confirmed that the PICaSSO correlated well with the histological evaluation (calculated through the Nancy and Robarts scores) and that this correlation was higher than the evaluation with Mayo endoscopic score (MES) and UC Endoscopic Index of Severity (UCEIS).^8^ In the current issue of the United European Gastroenterology Journal, Nardone et al. compared the rates of hospitalization, surgery, and medical therapy escalation in 302 UC patients achieving combined endoscopic‐histologic remission at 12 months or endoscopic remission alone using the high definition VCE PICaSSO score.^9^ Interestingly, no difference in the clinical outcomes was found between the endoscopic remission defined according to the PICaSSO score (≤3) and the endoscopic remission combined with the histological one (calculated both through the validated Nancy score and the Robarts histological index). Conversely, when endoscopic remission was measured by MES or UC Endoscopic Index of Severity (UCEIS), a lower rate of clinical events was recorded in the combined histology and endoscopy group at 6 (*p* = 0.04) and 12 months (hazard ratio 0.30, 95% CI 0.12–0.75, *p* = 0.02), respectively. According to these results, the PICaSSO score appears more rigorous than MES and UCEIS, and predicts clinical outcomes as well as the combination of endoscopy and histology, raising doubts about the need for histological evaluation and the search for a deeper remission than endoscopic. The use of the PICaSSO score may have a significant impact in clinical practice as it makes the collection of biopsies not necessary, reducing thus the time of the procedures and the risk of complications such as bleeding and perforation, as well as a significant reduction in costs. Moreover, it may allow a faster decision‐making time providing useful information for the management of the patient in real time. Beside these promising aspects, some considerations need to be made. Firstly, direct evidence on the alternative role of VCE compared to standard endoscopy + histology coming from a randomized clinical trials is still missing. Secondly, the PICaSSO score is calculated by VCE data from a specific endoscope, which is not available in all centers, limiting its wide use. Evidence on the validity of the PICaSSO scoring system with colonoscopes that use other methods of VCE (e.g., Blue‐light imaging, Linked color imaging, and Narrow‐band imaging) needs further confirmation. Furthermore, this technique requires a standardized training before it can be used by non‐experts. Since endoscopic biopsies are performed for multiple reasons including assessment of disease activity (i), colorectal cancer screening (ii), and exclusion of infections (e.g. cytomegalovirus infection) (iii), the use of a VCE approach without biopsies may be probably not appropriate in all patients.^10^ In the near future, technological progress will increasingly help clinicians to tailor diagnostic and monitoring procedures in patients with inflammatory bowel diseases, by optimizing time and resources. This study definitively opens the way in this direction.

## CONFLICT OF INTEREST

Ferdinando D’Amico declares no conflict of interest. Alessandra Zilli declares no conflict of interest. Gionata Fiorino received consultancy fees from Ferring, MSD, AbbVie, Takeda, Janssen, Amgen, Sandoz, Samsung Bioepis, Celltrion.

## AUTHORS’ CONTRIBUTIONS

Ferdinando D’Amico wrote the article. Alessandra Zilli and Gionata Fiorino critically revised the manuscript. The manuscript was approved by all authors.

## Data Availability

Data sharing not applicable to this article as no datasets were generated or analyzed during the current study.
